# Exploring schizophrenia spectrum psychopathology in borderline personality disorder

**DOI:** 10.1007/s00406-019-01039-4

**Published:** 2019-07-09

**Authors:** Maja Zandersen, Josef Parnas

**Affiliations:** 1 Mental Health Centre Glostrup, University Hospital of Copenhagen, Broendbyoestervej 160, 2605 Broendby, Denmark; 2 Institute of Clinical Medicine, University of Copenhagen, Copenhagen, Denmark; 3 Center for Subjectivity Research, University of Copenhagen, Copenhagen, Denmark

**Keywords:** Nosology, Epistemology, Differential diagnosis, Identity disturbance, Feelings of emptiness, Self-disorders

## Abstract

We have previously argued that the current borderline personality disorder (BPD) diagnosis is over-inclusive and clinically and conceptually impossible to distinguish from the schizophrenia spectrum disorders. This study involves 30 patients clinically diagnosed with BPD as their main diagnosis by three BPD dedicated outpatient treatment facilities in Denmark. The patients underwent a careful and time-consuming psychiatric evaluation involving several senior level clinical psychiatrists and researchers and a comprehensive battery of psychopathological scales. The study found that the vast majority of patients (67% in DSM-5 and 77% in ICD-10) in fact met the criteria for a schizophrenia spectrum disorder, i.e., schizophrenia (20%) or schizotypal (personality) disorder (SPD). The schizophrenia spectrum group scored significantly higher on the level of disorders of core self as measured by the Examination of Anomalous Self-Experiences Scale (EASE). The BPD criterion of “identity disturbance” was significantly correlated with the mean total score of EASE. These findings are discussed in the light of changes from prototypical to polythetic diagnostic systems. We argue that the original prototypes/gestalts informing the creation of BPD and SPD have gone into oblivion during the evolution of polythetic criteria.

## Introduction

Borderline personality disorder (BPD) is one of the most frequently used clinical diagnoses in both US and Europe. According to DSM-5 [1], the prevalence of BPD among inpatients is 20% and thus approaching the level of schizophrenia [2]. In contrast, the percentage of hospitalized patients diagnosed with personality disorders (PD) with DSM-II and ICD-8 was 1% and 4.6% in New York and London, respectively [3]. These dramatic changes in the proportion of diagnostic classes may reflect changes in incidence or help-seeking patterns but are most likely determined by altered diagnostic criteria and their clinical application.

The BPD category has evolved from very mixed conceptual and clinical sources, including sub-psychotic forms of schizophrenia and extraverted, dramatic patients with intense, but unstable personal relations [4]. When entering the DSM-III [5], BPD was separated from its evolutionary “twin”, schizotypal personality disorder (SPD; schizotypal disorder in ICD-10 [6]), formerly often denoted as “borderline schizophrenia”. In contrast to the BPD diagnosis, the schizotypal diagnosis is today rarely used and mainly in psychiatric facilities with research interest in schizophrenia [2, 7, 8]. In a detailed historical, conceptual, and empirical review [4], we have argued that the division of the borderline group into BPD and SPD was not entirely justified, and that the BPD category today is over-inclusive and both clinically and conceptually difficult to differentiate from the schizophrenia spectrum disorders. In a separate study [9], we have argued that the BPD criteria of “identity disturbance” and “chronic feelings of emptiness” are multi-layered phenomena which in their basic aspects of structural change of experience were both originally ascribed to the schizophrenia spectrum [10].

The purpose of the present study was to examine in depth the psychopathological profiles of patients considered to be suffering from BPD by psychiatrists at facilities specifically dedicated to the treatment of BPD. Our primary interest was to explore potential links to schizophrenia spectrum psychopathology. Specifically, we wanted to examine in more detail the possible connection between the BPD criteria of “identity disturbance” and “chronic feelings of emptiness” and the disorders of “core self” described in recent research literature as characteristic trait features of the schizophrenia spectrum [11–16].

## Method

### The sample

The patients were recruited from three university-affiliated outpatient clinics of the capital region of Denmark dedicated to the treatment of BPD. Originally, the patients came to those treatment facilities referred by other psychiatrists who considered them to suffer from BPD. This diagnosis was then confirmed at the outpatient clinic using a clinical interview or in some cases the Structured Clinical Interview for DSM-IV Axis II Personality Disorder (SCID-II) [17]. Patients were initially informed about the project by the treating psychologists or psychiatrists or, in some cases, directly by the first author who joined a group therapy session to invite patients to participate. Our colleagues from the treatment facilities were asked to select only patients diagnosed with BPD as their main diagnosis and without a clinical significant alcohol or substance abuse. A total sample of 43 patients agreed to participate in the study. However, 11 patients did not show up for the interview despite several appointments and two additional patients dropped out after initial contact. The final sample consisted of 30 patients (70% of the original group). The study was approved by the Danish National Committee on Health Research Ethics and informed written consent was obtained from all participants after receiving full explanation of the study.

### Assessment

The psychopathological and diagnostic interview employed a composite of different scales originally developed for the Copenhagen High-Risk Study [18, 19] and subsequently modified for the Linkage Study [20] and for several psychopathological studies at our department [11, 16, 21–23], which were all under the direction of the second author (JP; a senior consultant clinical and research psychiatrist). This instrument comprises a detailed psychosocial history and illness evolution, family history, the OPCRIT checklist [24], which is derived from the Present State Examination, supplemented with certain items from the Schedule for Affective Disorder and Schizophrenia (SADS-L) [25], a score sheet for ICD-10 and DSM-5 criteria for personality disorders (based on the Structured Clinical Interview for DSM-5 Personality Disorders (SCID-5-PD) [26], the perceptual section of the Bonn Scale for the Assessment of Basic Symptoms (BSABS) [27], and a Mental Status Examination. In addition, the Examination of Anomalous Self-Experiences Scale (EASE) [28] was included in the assessment. Since all patients were aware of their clinical BPD diagnosis, the interviewer went through each BPD criterion, recording the patients’ own opinion and interpretation.

The interviews took place at our department, the BPD treatment facility or the patients’ home. The interviews were of average 5 h duration (3.5–6) and were usually split into two or three sessions. All interviews but one were video recorded. Following the interview, a narrative summary was made of all sections of the interview (5–9 pages). The interview was semi-structured and conversational, encouraging faithful self-description according to standard phenomenological principles [29–32]. Rating of a criterion was never based on a simple “yes” or “no” answer but required examples provided by the patient. The structured element of the interview consisted in the obligation of covering all items of the assessment. The interviews were performed by the first author (MZ), an experienced clinical psychologist specialized in psychotherapy of psychotic patients and officially certified instructor of the EASE interview [28]. Prior to the study, MZ was tested for interrater reliability on the EASE scale with a senior consultant researcher (Dr. Lennart Jansson) with the overall value of Kappa 0.77. The interrater reliability between MZ and JP on the composite interview schedule prior to the study was of Kappa 0.85.

### The diagnostic process

Research diagnoses were allocated according to DSM-5 and ICD-10. The interviewer allocated the diagnosis on the basis of the interview combined with subsequent video reviewing of the material. In cases of uncertainty about crucial psychopathological phenomena (e.g., transient psychotic episodes vs enduring psychosis), MZ and JP jointly evaluated extracts of video recordings or made a joint extra interview with the patient [in 40% of the cases (*N* = 12)]. The final diagnosis was made at a consensus meeting between MZ and JP. A random sample of five interview summaries was independently diagnosed by external psychiatrist Dr. Peter Handest (Psychiatric Centre North Zealand), who arrived at the same diagnoses as the consensus diagnoses.

### Statistical analyses

In data analysis, all items were used “conservatively”, i.e., items scored as “doubtfully present” were recoded as “absent”. We used ANOVA to compare EASE levels across the diagnostic groups: non-schizophrenia spectrum (BPD and “other diagnoses”), schizophrenia, and SPD. Correcting for multiple testing, we used Scheffe’s test. Since the schizophrenia and SPD groups had similar scores, we merged these into a schizophrenia spectrum group and used the Student’s *t* test to compare this group with the non-spectrum group. The assumption about normal distributed residuals was tested with Shapiro-Wilk test and it was fulfilled. For testing correlations between the EASE, selected items from the interview (“identity disturbance” and “feelings of emptiness”) and the BPD and SPD scales, we used non-parametric Spearman’s correlations. All analyses were conducted using the STATA version 14.1 and SPSS version 25. Significance level was set to 0.05.

## Results

The socio-demographic data, treatment information, and duration of psychopathology appear in Table 1. All patients had a clinical main diagnosis of BPD and 37% had one or more clinical comorbid diagnoses of anxious (avoidant) PD, dependent PD, anankastic PD, paranoid PD, recurrent depression, post-traumatic stress disorder, attention-deficit/hyperactivity disorder or adjustment disorder. A majority (70%) had previously been diagnosed with a non-BPD diagnosis, mostly affective disorders and anxiety/stress-related disorders, and 57% had been hospitalized.

**Table 1:** Descriptives

	Mean (SD)/frequency	Range
*N*	30	
Gender, F/M	28/2	
Age, years	30 (8.0)	[18, 52]
Unmarried	27%	
Educational level		
Primary school or less	33%	
High school	30%	
College	17%	
Started university	13%	
Finished university	7%	
Social isolation	33%	
≥ 1 previous non-BPD diagnosis	70%	
Previously hospitalized	57%	
Age at first symptom, years	13.4 (8.6)	[6, 50]
Number of ambulatory treatments	4 (3.5)	[1, 20]
Duration of current treatment		
One month or less	10%	
1–6 months	20%	
7–12 months	37%	
> 12 months	33%	
Medication		
Antidepressants	37%	
Antipsychotics	10%	
Lamotrigine	7%	
Benzodiazepines	3%	

Social isolation: “fewer than two intimate non-family relations”

Table 2 shows the frequency of DSM-5 and ICD-10 BPD and SPD research criteria in descending order. The most frequent criteria were the SPD criteria of “inappropriate/constricted affect” and “unusual perceptual experiences”, whereas the least frequent criteria were the BPD criteria of “impulsivity” and “intense and unstable relationships”. An ICD-10 BPD diagnosis (i.e., “emotionally unstable PD, borderline type”) requires at least three criteria of “impulsive type” (IMP) of “emotionally unstable PD” AND at least two “borderline type” (BOR) criteria before the BPD diagnosis can be made. We also calculated the internal consistencies of the BPD and SPD item sets. For DSM-5, Cronbach’s alpha was 0.448 (BPD) and 0.653 (SPD), and for ICD-10 0.534 (BPD) and 0.609 (SPD). Item sets with joined DSM-5/ICD-10 criteria only had a slightly improved alpha (0.507 for BPD; 0.666 for SPD). Cronbach’s alpha for the EASE scale was 0.880.

**Table 2:** Frequency of BPD and SPD criteria in the sample

BPD and SPD criteria	*N*	(%)
Inappropriate/constricted affect	23	(76.7)
Unusual perceptual experiences	23	(76.7)
Odd thinking and speech	21	(70.0)
Identity disturbance^a^	20	(66.7)
Suspiciousness/paranoid ideation	18	(60.0)
Ideas of reference (DSM-5 only)	16	(53.3)
Transient paranoid ideation/severe dissociative symptoms (DSM-5 only)^a^	15	(50.0)
Odd beliefs/magical thinking	14	(46.7)
Intense anger^a,b^	13	(43.3)
Social withdrawal	13	(43.3)
Affective instability/unstable mood^a,b^	12	(40.0)
Quasi-psychotic episodes (ICD-10 only)	12	(40.0)
Feelings of emptiness^a^	11	(36.7)
Ruminations without inner resistance (ICD-10 only)	11	(36.7)
Recurrent suicidal/self-mutilating behaviour^a^	11	(36.7)
Excessive social anxiety (DSM-5 only)	9	(30.0)
Efforts to avoid abandonment^a^	7	(23.3)
Odd behavior	7	(23.3)
Difficulty in maintaining actions that offer no immediate reward (ICD-10 only)^a,b^	5	(16.7)
Quarrelsome behavior (ICD-10 only)^a,b^	2	(6.7)
Impulsivity^a,b^	1	(3.3)
Intense and unstable relationships^a^	1	(3.3)

DSM-5 BPD requires at least 5 criteria present. ICD-10 BPD requires at least 3 criteria of “emotionally unstable PD, impulsive type” (IMP) and at least 2 criteria of “emotionally unstable PD, borderline type” (BOR). The diagnostic threshold for SPD is at least 5 criteria in DSM-5 and at least 4 criteria in ICD-10

^a^BPD criterion

^b^IMP criterion in ICD-10

The research diagnoses of DSM-5 and ICD-10 appear in Table 3. Crucially, in our diagnostic assessment, only three patients were diagnosed with DSM-5 BPD, and only one patient was diagnosed with ICD-10 BPD. The most frequent diagnosis was SPD (*N* = 14 for DSM-5; *N* = 17 for ICD-10). Six patients (20%) received a DSM-5 and ICD-10 diagnosis of schizophrenia. It deserves mentioning that if we disregard the distinction between IMP and BOR criteria in ICD-10, then five patients had at least five ICD-10 “emotionally unstable PD” criteria present. However, four of these patients also had at least four (threshold) ICD-10 SPD criteria and were diagnosed with ICD-10 schizophrenia (*N* = 1) and SPD (*N* = 3) according to the hierarchy of ICD-10.

**Table 3:** Research diagnoses and mean EASE scores

DSM-5	ICD-10					
	Schizophrenia	SPD (schizotypal disorder)	BPD (emotionally unstable PD, borderline type)	Other diagnoses^b^	Total	EASE^b^ [range]
Schizophrenia	6				6	19 [9, 26]
SPD		14			14	16.8 [5, 25]
BPD		2	1		3	9.67 [9, 11]
Other diagnoses^b^		1		6	7	5.57 [1, 19]
Total	6	17	1	6	30	13.9 [1, 26]
EASE^a^ [range]	19 [9, 26]	16.12 [5, 25]	9	3.33 [1, 5]	13.9 [1, 26]	

^a^Mean score of main items (*N* = 57) scored dichotomically (present/not present)

^b^This group comprised patients with other PDs than BPD/SPD (specified, other or mixed), adjustment disorders, and other psychotic disorder

The mean number of BPD criteria in the sample was 2.8 (DSM-5) and 3 (ICD-10), ranging from 0 to 7. The mean number of SPD criteria was 4.8 (DSM-5) and 4.7 (ICD-10), ranging from 0 to 8. Thus, criteria from both diagnostic categories were quite prevalent in the sample and present in combination in several individual patients across diagnostic categories. For instance, the mean number of BPD criteria among the schizophrenia patients were 2.8 (DSM-5) and 2.5 (ICD-10).

Table 3 also displays the mean EASE-total score. In both DSM-5 and ICD-10, patients with schizophrenia and SPD had significantly (*p *< 0.001) higher levels of self-disorders than the non-spectrum group. There were no significant differences in EASE score between the schizophrenia and SPD group in both diagnostic systems. For DSM-5, mean EASE-total score for the schizophrenia spectrum group was 17.45 (SD 6.04) and for the non-spectrum group 6.8 (SD 5.39). For ICD-10, the respective means were 16.87 (SD 6.03) and 4.14 (SD 2.61). All these scores are very similar to those of other studies [14, 23, 33].

The BPD criterion “identity disturbance” was significantly correlated with EASE-total score (*r *= 0.680, *p *≥ 0.001) and EASE domains (1), (2), (3), and (5) (*r *= 0.539, *p *= 0.002; *r *= 0.665, *p *< 0.001; *r *= 0.470, *p *= 0.009; *r *= 0.640, *p *< 0.001) and the joined DSM-5/ICD-10 SPD item set (*r *= 0.401, *p *= 0.028) but not with the joined BPD item set when excluding the identity criterion. The EASE domains are: (1) cognition and stream of consciousness, (2) self-awareness and presence, (3) bodily experiences, (4) demarcation/transitivism, and (5) existential reorientation [28]. The BPD criterion “chronic feelings of emptiness” was significantly correlated only with EASE domain (2) and (5) (*r *= 0.362, *p *= 0.050; *r *= 0.375, *p *= 0.041). There were no significant correlations between “chronic feelings of emptiness” and the joined DSM-5/ICD-10 BPD and SPD item sets. EASE-total score was significantly correlated with the joined SPD item set (*r *= 0.581, *p *= 0.001), but not with the joined BPD item set. The corresponding correlations for separate DSM-5 and ICD-10 item sets were similar to the joined item sets. There were no significant correlations between age and educational status on the one hand and the BPD and SPD item sets on the other. The EASE total score was uncorrelated with educational level but significantly and negatively so with age (*r *= 0.616, *p *< 0.001).

### Clinical case illustrations

We here present two case illustrations. Case 1 illustrates a typical patient who is not clearly psychotic and who may present differential diagnostic difficulties.

### Case 1

Female patient, 23 years old, has attended BPD treatment for 8 months. Since primary school, she has had different jobs from which she was fired due to conflicts with her bosses. She is now a university student. As a child, she was often in a fight, and she describes herself as being “hysterical”, “weird”, and “eccentric”. She has always felt that people stare at her on the street but has concluded that this is because she is so “weird”. She complains about lacking an identity and missing a “position in life” and describes how she modulates herself extensively according to her social environment. Her social engagements are limited and she spends most time alone. She generally finds it difficult to trust other people. At home, she may feel the presence of another person and she often sees silhouettes of people. She episodically experiences her body as nothing but “a skeleton with muscles and skin”. When looking in the mirror, she may become absorbed in speculations concerning if she would be able to recognize herself on the street.

She complains about a permanent chaos of feelings that may intensify into episodes of what she terms “a firestorm” of feelings where she cannot identify any feelings. Sometimes, this leaves her in a numb state of emptiness. At other times, she cuts herself to “escape inner chaos”—a behavior which she refers to as “impulsive”. She also describes a painful feeling of “complete indifference toward the world”, occasionally accompanied by the world appearing unreal and “inaccessible” as if she is enclosed behind a wall of glass. She then visually perceives the world in an un-colored tone.

She has several simultaneous layers of thoughts and may speculate for hours about physics, linguistics, social conventions, etc. At the same time, she reflects about herself having these thoughts and compares this “thinking about thinking” to the experience of seeing oneself in a mirror and so forth. Occasionally, inappropriate or unpleasant vivid inner visual scenarios pop up, e.g., of a bloody accident. She describes such thoughts as being more vivid than normal thoughts, appearing as a sort of “parallel reality”.

During the interview session, the patient appears with an un-modulated affect. Her mood is neutral. Her thinking is circumstantial and her language stilted. She often describes herself in a third person perspective. There is a stereotypic use of specific phrases and she mixes Danish and English in a profound and superficial way. The patient fulfills both ICD-10 and DSM-5 criteria of schizotypal (personality) disorder (e.g., social isolation, constricted affect, perceptual disorders, obsessive ruminations, derealization/depersonalization, formal thought disorders, and ideas of reference).

### Case 2

Female patient, 24 years old, recently referred to a BPD treatment facility. She lives with her boyfriend and has been on a sick leave for 3 years. She has no friends, stating “I do not even know what it means to have a friend”. She is very ambivalent and let her boyfriend decide on everything.

She describes being rather anxious as a child. When walking to school she felt that someone was following her and at home, she had an unpleasant feeling of a male person watching her from outside of the home. She felt “unhappy” and as a 12-year-old, she was treated medically for depression. A few years later, she began hearing the voice of her dead grandfather telling her not to be afraid and she felt comforted by this. As a teenager, she began cutting herself several times a day on arms, legs, stomach and chest due to an experience of inability to feel anything, including herself. She had a suicide attempt as 19-year-old. Since then, she has primarily had numerous contacts in somatic care due to feelings of “heaviness” in body parts or pain in her “bones”.

She experiences a general lack of feelings and an almost complete lack of pleasure and desire stating that “I probably lack the pleasure of life”. Sometimes, a feeling may pop up or disappear seemingly without any reason, as if switching on or off a contact. She explains how everything she feels or thinks “probably” comes from her brain and she experiences it as if a “crane” suddenly pulls up a thought or a feeling from a tub filled with “everything” and then dumps it in her head.

She has a “constant chaos of sound” in her head. For instance, she has a loop of a girl saying “you are not allowed to lie” and a loop of her mother’s voice saying “that sounds good”. The patient does not feel it has anything to do with her. She also hears her own thoughts aloud and describes having so many thoughts “flying around” that she cannot identify any coherent content. She avoids social contact since in the presence of other persons, she feels they can hear her thoughts and she wants to keep “this mess of thoughts” to herself. Asked for how long she has had such experiences, she tells that as a child she feared being bullied because of her thoughts, believing that other pupils could hear them. This literally made her stay several meters away from her classmates. Today, she has a constant feeling of being watched or followed and she almost exclusive leaves her apartment to walk her dog. At home, she always draws down the curtains.

Asked about impulsivity, she describes how she on a summer day felt like taking a swim but were too anxious to leave her apartment. She then “impulsively” put up a paddling pool in her second floor apartment and sat in it with her dog, resulting in a damage of floor and furniture. When asked what led her to do this, she replied “I couldn’t see why not”.

During the interview, the patient avoids eye contact and speaks in an almost whispering tone. Her mood is neutral but her emotional expression is flat and her thinking is formally disturbed including severe neologisms and private logic. Sometimes, she is rocking in a somewhat stereotypic manner. The patient fulfills criteria for schizophrenia in both ICD-10 and DSM-5 (e.g., delusions of thought broadcasting, auditory verbal hallucinations, and negative symptoms).

## Discussion

We will first address the limitations of this study. Clearly, the sample size is small. However, this size must be seen in the light of a very thorough psychopathological assessment involving experienced psychologists and psychiatrists. This is perhaps also an issue because of its limited generalizability and ecological comparability with other studies. Thus, although we cannot exclude an observer bias specific to our research group, we have tried to make a maximal effort to arrive at a thorough self-description of psychopathology including all diagnostically relevant criteria. Whereas such assessment approach was common in psychiatric research until 1990’s, contemporary data collections and diagnostic assessments are often performed with self-report questionnaires or brief structured interviews often made by “for-the-purpose-trained” interviewers without any background in psychopathology [34–36]. We, and others, have addressed in-depth these epistemological issues elsewhere [37–42]. Another limitation is a potential selective bias of the assessed patients. We have no information about potential differences between this group and those who chose not to volunteer. Of the 43 patients, who initially agreed to participate, only 30 completed the study. There were no differences in socio-demographic or clinical characteristics between these two groups. Finally, an obvious limitation of a cross-sectional clinical study is that the patients find themselves in different stages of their illness. While we acknowledge these limitations, we believe that our patients, recruited from 3 different BPD clinics, are in fact representative of BPD patients as they are diagnosed in the clinical setting today.

The study’s main finding is that in a sample of 30 clinically diagnosed BPD patients, the vast majority of patients met criteria for a schizophrenia spectrum disorder, i.e., SPD or schizophrenia (67% in DSM-5 vs. 77% in ICD-10). This was anticipated by Meehl who in 1989 offered the “rash prediction” that “most of the patients called borderline are [in fact] schizotypes” [43, p. 949]. The second major finding is that only a few (3 in DSM-5 vs. 1 in ICD-10) fulfilled definite criteria for BPD. Thus, schizophrenia spectrum psychopathology is prevalent in clinically diagnosed BPD patients and perhaps also considered as compatible with a BPD diagnosis by psychiatric clinicians.

With respect to psychotic symptoms, 20% of our sample met criteria for schizophrenia and another 40% for “quasi-psychotic episodes” (SPD criterion in ICD-10). Five patients had psychotic symptoms that were more articulated than at a “quasi-psychotic” level, yet still failing to meet the criteria for schizophrenia. From a clinical perspective, we can consider these patients as falling in between the diagnostic criteria of SPD and schizophrenia. Such patients, as well as a substantial proportion of our schizotypal patients, would have received a schizophrenia diagnosis in the ICD-9 [21, 44, 45]. Unfortunately, psychotic symptoms are in the clinical setting often under-detected or even ignored when detected. A recent Norwegian study [46] found that the presence of apparently “neurotic” symptoms in the initial phases of schizophrenia resulted in a disregard of the underlying psychotic psychopathology. Such neurotic symptomatology may be more prevalent among help-seeking (vs. non-help-seeking) schizophrenia spectrum individuals [47]. Another study [48] found that in 56% of cases where clinicians recorded psychotic symptoms, this resulted in either a non-psychotic diagnosis or no diagnosis at all. A Danish register-based study [49] found that among patients diagnosed with ICD-10 BPD (*N* = 10,876) between 1995 and 2010, approximately 70% (equivalent to the percentage of our sample) had been diagnosed with a non-BPD diagnosis as their first-ever diagnosis and 55% received a non-BPD diagnosis as their latest diagnosis.

The BPD diagnosis first entered the diagnostic manuals of DSM-III and later of ICD-10. It is worth emphasizing that both manuals involved a fundamental epistemological shift from a diagnosis based on narrative descriptions with prototypical and conceptual considerations to the so-called a-theoretical, polythetic categories defined by a sufficient number of specific criteria. These categories are insufficiently articulated in terms of their phenomenological quiddity (i.e., their “whatness”), and the criteria are typically poorly defined [9].

The input on the pre-DSM-III borderline concept came mainly from three sources: (1) the clinical and psychotherapeutic notion of sub-psychotic cases of schizophrenia originally described by Bleuler as “latent schizophrenia” [10] and in the modern era as, e.g., “pseudoneurotic schizophrenia” [50], “borderline schizophrenia” [51], “schizotypal disorders” [52, 53] or “Hoch-Polatin syndrome” [54]. This psychopathological Gestalt comprised subtle Bleularian fundamental symptoms such as disorders of expressivity and affectivity, formal thought disorder, ambivalence, experiential ego-disorders, and a variety of psychosis-near dis-integrative features. (2) Another Gestalt arose mainly from psychotherapeutic practice and described extroverted, dramatic patients with intense but fluctuating interpersonal relationships, shifting between idealization and devaluation and being problematic to manage and treat in a psychotherapeutic setting [55, 56]. (3) A third source was Kernberg’s concept of borderline personality organization [57, 58], which is a structural-dynamic concept describing, e.g., a diffusion of identity and a specific pattern of defense mechanisms such as denial, splitting, and projective identification. This latter concept was a trans-diagnostic dimension applicable to such different categories as schizoid (and presumably schizotypal), paranoid, hypomanic, narcissistic, and anti-social personalities and different psychosis-near disorders [4]. Thus, Kernberg’s concept was not intended for polythetically defined categories.

It was upon these very different conceptual sources that the American Psychiatric Association initiated a study with the aim of developing polythetic criteria for borderline diagnoses. The study [59] operated with a set of “schizotypal” items (SPD) derived from the concept of borderline schizophrenia and a set of “unstable personality” items (BPD), reflecting a mixture of the unstable Gestalt with selected input from Kernberg’s theory. The construction of polythetic criteria was believed to reflect these different Gestalts. A questionnaire was sent to American psychiatrists who rated one borderline patient and one patient acting as a control. Approximately 75% of these patients came from private practices. The study found that upon cut-off scores that best discriminated the two categories, 54% of the patients still fulfilled criteria for both diagnoses. This study formed the basis of separating the borderline concept into BPD and SPD in the DSM-III.

Based on our previous work, we argue that since 1980, the founding prototypes and the original psychopathological insights that imbued the creation of single polythetic criteria have gone into oblivion [4, 9]. Criteria have been modified and have undergone unnoted semantic drifts [4], and a ninth BPD criterion was added to the DSM-IV [60], i.e., “transient, stress-related paranoid ideation or severe dissociative symptoms”. Near-psychotic symptoms are, therefore, present as diagnostic criteria in both BPD and SPD in the DSM-5. These developments have made the differentiation of the diagnoses fuzzy and heavily dependent on the detection and registration of the schizophrenic fundamental symptoms. Several of these symptoms are included as SPD criteria, i.e., subtle forms of formal thought disorder, inadequate forms of rapport and affect, eccentricity, and mannerisms. Unfortunately, clinicians and researchers no longer pay careful attention to those features [61, 62], and their expressive nature make them impossible to be assessed through self-report questionnaires and structured interviews.

We will now turn to differential diagnostic difficulties concerning the specific BPD criteria of “impulsivity”, “identity disturbance”, and “chronic feelings of emptiness”. It is remarkable that only 1 patient in our sample met the BPD impulsivity criterion. Very few patients mentioned impulsivity spontaneously. When interrogated systematically, some patients affirmed being impulsive, yet closer examination revealed that this was not the case. For example, one patient found herself to be impulsive in the sense that she had “a chronic impulse to withdraw from social contact”. Impulsivity as a personality trait refers to a tendency to act and react faster and with a shorter reflection time compared to normatively appropriate behavior. Such tendency, or trait, must manifest itself in different situations across the span of life and not only pop up as an unexpected instance of, e.g., self-mutilating behavior (see case 1 above). Impulsivity may resemble, but must essentially be distinguished from, chaotic or incomprehensible behavior caused by a disintegration and chaos of inner life, which is characteristic of the schizophrenia spectrum. In case 2 (see above), the patient describes how she “impulsively” puts up a paddling pool in her apartment. Considering also her psychopathological Gestalt which includes a severe disintegration of the self (see below), this particular action illustrates bizarre behavior in schizophrenia spectrum disorders, designated by Minkowski as “autistic activity” [63], by Blankenburg as “crisis of common sense” [64], and by Conrad as “crazy actions” [65].

The most prevalent BPD criterion was “identity disturbance”, which DSM-5 defines as a “markedly and persistently unstable self-image or sense of self” [1, p. 663], which is phenotypically manifest as, e.g., “shifting goals, values, and vocational aspirations” [1, p. 664]. Disorders of self, including disturbance of identity, have historically been described as fundamental features of the schizophrenia spectrum and have in the last decades been empirically shown to hyper-aggregate in schizophrenia and SPD [66]. This disturbance of the sense of self in schizophrenia was addressed in the DSM-III “Glossary of Technical Terms” [5]. However, neither the DSM-5 nor the ICD-10 operate with the notion of self-disorders so fundamental to schizophrenia spectrum conditions. As we have argued elsewhere [9], it may be differential diagnostically useful to distinguish between a “narrative” and a “core” level of selfhood. The narrative level concerns descriptive features such as personality traits, temperament, and cognitive dispositions, i.e., our personal identity. Disorders of self or identity may articulate itself on a narrative level as uncertainty about one’s preferences, goals, and values (as in the DSM-5) (see also case 1 above). The notion of “core” self, however, refers to the structure of our experience, which normally is characterized by an automatic and pre-reflective first-personal articulation of experience with a permeating sense of self-presence. The core self refers to the formal aspects of “how” the elusive sense of subjectivity at all comes to be. Disturbances at this core level may include a disorder of the first-person perspective, a diminished sense of self-presence, and a fragile sense of psychophysical unity and self-world demarcation. Such disturbances are clearly present in both of the above case illustrations despite their apparent differences in symptomatology. Disturbances of the core self also often manifest at a narrative level of selfhood. This is clearly visible in the significant correlation between the identity disturbance criterion and the EASE-scale. In fact, it is often at the narrative level that disturbances of core self first signal themselves [9].

In the pre-DSM-III literature, identity disturbance and feelings of emptiness were tightly interwoven, with feelings of emptiness being one way of subjectively experiencing the disturbance of identity or of core self [9]. This psychopathologically important connection has been erased in contemporary diagnostic systems. The SCID-5-PD contains only a single question pertaining to feelings of emptiness, namely “do you often feel empty inside?” [26]. In the present study, we did not find feelings of emptiness to be significantly correlated with the BPD identity disturbance criterion. However, both criteria correlated significantly with EASE Domain 2 (targeting first-person perspective, self-presence, and world immersion) and Domain 5 (targeting existential orientation) [28].

In conclusion, this study of 30 clinically diagnosed BPD patients reveals that when polythetic criteria are carefully applied on the basis of a comprehensive psychiatric assessment involving senior level psychologist and psychiatrist, a substantial majority of these patients fulfill in fact the diagnostic criteria of the schizophrenia spectrum. Similar results have recently been reported for patients with obsessive–compulsive disorder (OCD) [67]. This seems to indicate that the original psychopathological prototypes and the conceptual understanding of the single symptoms have gone into oblivion and that clinicians are, therefore, liable to form their own (idiosyncratic) prototypes. There is a risk that such prototypes become associatively triggered by single, emblematic symptoms (“self-mutilation → borderline”) and not associated with a careful differential diagnostic assessment of all criteria [68]. It may be the case that the BPD diagnosis of both of the above clinical case illustrations were dependent on self-mutilation, identity disturbance and emptiness without considering the structural disorders of the core self and the expressive features of schizophrenia spectrum. This is not only a differential diagnostic problem but also an issue with important implications for treatment. Today, BPD and schizophrenia spectrum disorders are usually treated in highly specialized treatment facilities with diverse treatment approaches depending on the diagnosis. There is a risk that patients receive yearlong treatment based on a wrong diagnosis and with limited results. Although a comprehensive psychopathological assessment such as the assessment in this study including the EASE interview is perhaps not feasible as a standard in a daily clinical routine, we believe that a more thorough assessment that also includes important experiential features and the diagnostic expressive features of the schizophrenia spectrum will improve differential diagnosis considerably. Such improvement will also necessitate a more thorough psychopathological training.
